# Cancer and how the patients see it; prevalence and perception of risk factors: a cross-sectional survey from a tertiary care centre of Karachi, Pakistan

**DOI:** 10.1186/s12889-019-6667-7

**Published:** 2019-04-01

**Authors:** Saira Saeed, Javaid Ahmad Khan, Nousheen Iqbal, Sana Irfan, Alviya Shafique, Safia Awan

**Affiliations:** 1 Jinnah Sindh Medical University, Karachi, Pakistan; 2 Department of Medicine, Aga Khan University Hospital, Karachi, Pakistan; 3 Jinnah Medical and Dental College, Aga Khan University Hospital, Karachi, Pakistan; 4 Aga Khan University Hospital, Karachi, Pakistan

**Keywords:** Cancer, Prevalence, Myths, Risk factor

## Abstract

**Background:**

The incidence of cancer is rising but data available regarding prevalence of cancer and patient perception of the disease in Pakistan is limited. It is difficult to deal with Cancer if the main causes are negligence towards risk factors and bizarre myths. This study was aimed to investigate common cancer presentations at a government sector hospital and to gain insight into patient knowledge of the disease.

**Methods:**

This was a cross-sectional study conducted on cancer patients from Jinnah Postgraduate Medical Centre. A self-made questionnaire was used to assess the norms related to cancer prevalence in our society, associated myths, and the most common risk factors per them.

**Results:**

A total of 402 participants consented to participate in the study (mean age 42.3 ± 15.07 years), 204(50.7%) were females and 190(47.3%) were illiterate. Biomass exposure was found in 147(37%), drug abuse in 132(33%) and smoking in 63(16%). We found 103(25.6%) had positive family histories of cancer. The most common primary tumor site was breast for females 98(48%) and Head and neck 66(33.3%) for males. Patients considered fate 328(82%), gutka 284(71%) and injuries 282(70%) as the most common causes for cancer; while 222(55.5%) considered black magic and 236(58.75%) considered evil eye as a risk factor for cancer. Cancer treatment caused significant financial stress in 376(93.5%) patients.

**Conclusion:**

Breast and head and neck cancers were found to be prevalent among patients. It was noted that patients are negligent in daily life regarding the consumption of substances that commonly cause cancer. Individuals had diminished knowledge and majority linked cancer to unrelated causes and myths like black magic and fate. Almost all the patients complained of severe financial stress imposed by the disease.

**Electronic supplementary material:**

The online version of this article (10.1186/s12889-019-6667-7) contains supplementary material, which is available to authorized users.

## Background

Cancer is a disease whereby affected body cells grow uncontrollably and deprive normal body cells of nutrients and appropriate function. The world health organization WHO fact sheet for February 2017 named Cancer as one of the major causes for deaths around the globe with 8.8 million deaths in 2015 resulting in 1 per every 6 deaths being cancer mediated [1]. It is estimated that around 140,690 cancer cases will be reported in 2019 [2, 3] and majority of these patients will continue to battle the disease lifelong.

A study done in 2012 regarding cancer prevalence in Pakistan, it was found that almost 63,415 males and 85,590 females were diagnosed with the disease [4]. The most common cause of cancer related deaths worldwide are lung cancers [1] while breast cancer continues to top the list with the maximum reported deaths in Pakistan [5]. There are numerous risk factors for cancer including hormonal, hereditary, metabolic, autoimmune etc. External causes of cancer include smoking, alcohol consumption, dietary imbalance (malnourished or obese), radiation or infections like Human Papilloma Virus (HPV), Hepatitis B Virus (HBV), Human Immunodeficiency Virus (HIV), H Pylori etc [6–8].

There is no nationwide cancer incidence figure available from Pakistan since the past 64 years. No Population-Based Study has been conducted; the only data that can be traced down is based on hospital registrations. Pakistan is the sixth most populous country in the world, where almost 80 million of the people (approximately 50% of the population) suffer from one of such chronic conditions [9]. It is necessary to monitor cancer thoroughly considering the rapid rate at which the disease burden is increasing in the country.

It is essential to study the exposure of patients to known cancer causing agents for successful cancer monitoring and prevention. The financial stress of Cancer diagnosis and treatment should also be studied to highlight their detrimental effect on the psycho-social aspect of the patient’s life. At the same time, numerous myths have emerged among the local population regarding causes of cancer. These myths should be noted and nullified so that the main contributory factors are identified correctly by the patient and they remain cautious with regards to them. The monetary demand of this disease exceeding the pocket of an average income based man coupled with diminished patient knowledge over the disease are all signs emphasizing on how vital it is to handle this disease as a national catastrophe.

The aim of the study is to determine knowledge regarding cancer and its associated risk factors among patients belonging to a low socioeconomic class and to highlight the most common types of cancers encountered in such a setting. The study also intends to note any unrelated causes that people commonly associate with cancer that deviate their attention from true ones. Furthermore, via this study we wish to focus on the psycho-social and monetary burden imposed by such a disease so that financial aid and behavioral therapies are encouraged for cancer patients.

## Methods

This was a cross-sectional study conducted on diagnosed cancer patients from Jinnah Postgraduate Medical Centre (JPMC) between October and November 2016. JPMC is a government-run, tertiary care hospital in Karachi, Pakistan where most of the patients come from a lower socio-economic status (SES). The ethical clearance for conducting this study was obtained from Chairman at JPMC. Informed consent was taken from each patient before proceeding with the questionnaire. In Pakistan numerous patients are not aware of their illness and aren’t informed that they have been diagnosed with Cancer. In such cases where the patient was unaware of his/her diagnosis, we made sure not to reveal the state of the disease to the patient and obtained our data either from the attendant or the patient himself, without using the word ‘cancer’ anywhere during the session.

A self-made questionnaire (Additional file 1) was made after thorough literature review and was pilot-tested on ten patients at the outpatient department of the Oncology Department, JPMC. For the purpose of a detailed analysis of cancer prevalence among various ethnic groups that exist in Pakistan, divisions like Sindhi, Balochi, Punjabi and Pathan were made. These groups were based on the home province and native language of the patient. Another group titled ‘others’ was designated for minor ethnicities that didn’t fit into any of these sub-groups which included Muhajir (Urdu speaking) Bengali and Hindko etc. To assess the socio-economic status of the participants and divide them accordingly, categories were made on the basis of the monthly income of the earning head of the family and were as follows:

1. Upper Class: Rs. 60,000 to Rs. 2,40,000
2. Middle Class: Rs. 20,000 to Rs. 60,000
3. Lower Class: Rs. 12,000 to Rs. 20,000
4. Poor Class: Rs. 6000 to Rs. 12,000
5. Extremely poor: Less than Rs. 6000
6. Unemployed

Education level was defined as follows:

1. Illiterate: without any primary education
2. Primary: First 1 to 8 years of schooling.
3. Secondary: From 9 to 12 grade.
4. Graduate: 2 years Post-secondary education.
5. Postgraduate: for 3 to 5 years aftergraduation.

Patients were interviewed by students taking part in the study. We included all patients who were either regularly visiting the outpatient department of Oncology, JPMC for cancer treatment or were admitted in ward. Those who refused to give consent; couldn’t communicate in Urdu/English and bedridden patients who were unable to converse were excluded from the study. Patients’ reports were read for details regarding disease progression and the treatment given. If someone didn’t have his/her report, the option ‘extent can’t be assessed’ was chosen.

### Statistical analysis

Descriptive analysis for demographic variables, cancer history and knowledge over the disease was performed; results were reported as numbers with percentages for quantitative variables, and mean ± standard deviation for all qualitative variables. SPSS 19.0 (SPSS Inc., Chicago: IL, USA) was used for data entry and analysis.

## Results

### Baseline demographics

A total of 402 participants consented to participate in the study. Mean age was 42.3 ± 15.07 years among which 204(50.7%) were females and more than half 310(77%) were married. Majority were illiterate (*n* = 190, 47.3%) and belonged to a poor socioeconomic class (*n* = 111, 27.6%). Around 336(83.6%) patients were aware of their cancer. By ethnicity 191 (47.5%) out of 402 participants belonged to various other ethnic groups including Muhajir, Memon, Gilgitetc, followed by Sindhi (*n* = 94, 23.4%) Balochi (*n* = 44, 10.9%) Pakhtun (*n* = 33, 8.2%). Punjabi was the least common ethnicity encountered (*n* = 40, 10%). Majority had Hypertension (HTN) (*n* = 56, 13.9%) and Tuberculosis (*n* = 34, 8.5%) was the second most prevalent co-morbid (Table 1).

**Table 1:** Baseline Demographics of study participants

Baseline Demographics		
Mean Age, in years	42.3 ± 15.07	
	Number (n)	Percentage (%)
Gender		
Male	198	49.3
Female	204	50.7
Marital status		
Married	310	77.1
Single	73	18.2
Divorced	3	0.7
Separated	3	0.7
Widowed	3	0.7
Ethnicity		
Sindhi	94	23.4
Balochi	44	10.9
Pathan	33	8.2
Punjabi	40	10
Others	191	47.5
Socioeconomic status		
Upper Class	3	0.7
Middle Class	47	11.7
Lower Class	91	22.6
Poor Class	111	27.6
Extremely Poor	91	22.6
Unemployed	59	14.7
Education level		
Illiterate	190	47.3
Primary	100	24.9
Secondary	73	18.2
Graduate	36	9.0
Postgraduate	3	0.7
The patient knows about his/her cancer	336	83.6
Data collected from the patient	265	65.9
Data Collected from Attendant	136	33.8
	Number (n)	Percentage (%)
Co-morbid		
DM^a^	22	5.5
HTN^b^	56	13.9
IHD^c^	13	3.2
COPD^d^	7	1.7
TB^e^	34	8.5
Hepatitis	19	4.7
Associated factors		
Smoking	63	15.7
Smokeless Tobacco	132	32.8
Alcohol	2	0.5
Biomass Exposure	147	36.6
Duration of factors’ consumption		
Duration of Smoking	15[10–30]	
Duration of Smokeless Tobacco	12 [5–20]	
Duration of Alcohol	17.5 ± 3.5	
Industrial Exposure Among Patients		
Industrial experience	66	16.4
Industry		
Garments/ Textile Industry	56	93.3
Metal Industry	2	3.3
Others	2	3.3
Designation		
Labour	46	78
Office work	3	5.1
Others	10	16.9

^a^Diabetes Mellitus, ^b^Hypertension, ^c^Ischemic Heart Disease, ^d^Chronic Obstructive Pulmonary Disease, ^e^Tuberculosis

#### Exposure to known risk factors

Among participants 63 (15.7%) were current smokers with an average duration being 15 ± 10 years. 132 (32.8%) of participants admitted the use of smokeless tobacco for an average duration of 12 ± 7 years. A total of 147 (36.6%) had biomass exposure and 66 (16.4%) had industrial exposure. Garments industry 56 (93.3%) was the most common industry patients were exposed to followed by metal industry reported in 2 (3.3%) patients. A total of 46 (78%) of the participants with industrial exposure were working as laborers.

#### Cancer statistics

The study revealed that a large number of participants 103 (25.6%) had a very strong family history of Cancer with most of them 76 (83.5%) having at least 1 relative suffering from cancer while 12 (13.1%) had 2 diseased relatives. Majority 76 (18.9%) of the relatives with cancer had died of it. Out of 103 relatives, 73 (18.2%) were currently or previously on treatment for their cancers. Out of 402 participants, a vast majority presented with Breast cancer (*n* = 100, 24.9%) making it the most common primary site of tumors followed by Head and neck (*n* = 89, 22.13%). Breast cancer was the most common cancer among females (48%) while majority males had head and neck cancer (33.3%) followed by blood cancer (26.3%) (Table 2).

**Table 2:** Cancer Statistics in study population

Family History		
	Number (n)	Percentage (%)
Relative		
Alive	15	3.7
Dead	76	18.9
Relatives who received treatment	73	18.2
Primary Site Of Tumor		
Type (Total = n)	Male (*n* = 198)	Female(*n* = 204)
Breast (100)	2(1.0%)	98(48%)
Head and neck (89)	66 (33.3%)	23(11.3%)
Blood (74)	52(26.3%)	22(10.8%)
GIT^a^ (47)	30(15.2%)	17(8.3%)
Bone (16)	11(5.6%)	5(2.5%)
Lung (9)	9(4.5%)	0
Bladder (3)	2(1.0%)	1(0.5%)
Prostate (2)	2(1.0%)	0
Ovary (13)	0	13(6.4%)
Other (38)	18(9.1%)	20(9.8%)
Not known/can’t assess (5)	3(1.5%)	2(1.0%)

^a^GIT: gastrointestinal tract

### Knowledge and psychological impact of cancer

(a)Knowledge Regarding Cancer Risk Factors.

The most common factor responsible for cancer reported by patients was fate (*n* = 328, 82%), followed by gutka consumption (*n* = 284, 71%), injuries (*n* = 281, 70%), betel nut (n = 281, 70%) and smoking (*n* = 275, 68.4%).A total of 236 (58.75%) considered evil eye as a risk factor for cancer too. Almost 200(49.8%) patients thought that family history contributed to cancer. A total of 124 (30.8%) females considered that the fact they stopped breastfeeding their children led to breast cancer development while 97 (24.1%) thought domestic violence could be a risk factor too (Table 3).

**Table 3:** Knowledge per Cancer and Psychological Impact of Cancer

Knowledge Per Cancer And Psychological Impact Of Cancer			
	Number (n)^a^	Percentage (%)	
Knowledge Per Known Risk Factors			
Do you think Smoking causes cancer?	275	68.4	
Do you think Betel nut causes cancer?	281	69.9	
Do you think Gutka causes cancer?	284	70.6	
Do you think Family History is a cause for cancer?	200	49.8	
Knowledge Per Misleading Risk Factors			
Do you blame Fate for your cancer?	328	81.6	
Do you blame Evil eye for your cancer?	236	58.7	
Do you blame Black magic for your cancer?	222	55.5	
Do you think Fever causes cancer?	222	55.2	
Do you think cancer is due to stopping breastfeeding?	124	30.8	
Do you think domestic violence led to your breast cancer?	97	24.1	
Psychological Impact Of Cancer			
On A Scale Of 0 (Min) To 5 (Max)			
	Scale	Number (n)^a^	Percentage (%)
How depressed do you feel?	3	118	29.4
How irritable have you become?	4	100	24.9
How much has your social life been affected?	3	130	32.3
How much has the financial burden increased?	5	125	31.3

^a^Number of individuals who responded yes

(b)Psychological and Financial Burden.

Of total 402 patients questioned, 93(23.1%) reported being depressed/discouraged due to their illness, 79(19.7%) had difficulty controlling their anger and only 2(0.5%) patients were satisfied with their overall quality of life. 376(93.5%) patients reported facing financial problems due to this cancer treatment.

## Discussion

This study was aimed to convey a comprehensive overview of cancer at a government sector hospital in Karachi, Pakistan. In this study we found, breast cancer most common in females and head and neck cancer among males primarily due to high consumption of cancerous substances among males like betel nut, *mainpuri, gutka* and cigarettes. The mean age of patients ranged more in the adult population as the risk for cancer is increase with increase age [10]. Worldwide, it has been seen that cancer is most frequently encountered in the male population. Out of the 35 sites studied in a research, 32 had a higher M: F ratio [11]. In our study, we encountered more females which can be explained by the high prevalence of breast cancer in Pakistan, where breast cancer makes up one-third of all the female cancers and its incidence is the highest overall in Asia [12].

Majority of the patients interviewed were illiterate and belonged to a poor socioeconomic class. Despite advancements, low SES remains a non-modifiable risk factor for various cancer types. A low SES and less education contribute to financial problems in disease treatment and late presentations or cessation of treatment in between due to monetary constraints. A European study stated that patients with less education had lower survival and much advanced disease at presentation [13]. When a comparison was drawn between patients with high and low levels of educations in a Uganda based research, it was found that the lower ones were diagnosed with advanced cancers more often [14].

Connections between co-morbidity and cancer have been well-established. Lee reported the prevalence of co-morbid in cancer patients to range as wide as between 0.4 to 90% [15] while Edwards et al. stated breast and prostate patients in the United States to have a similar incidence of co-morbid as the normal population [16]. For our study, HTN has been seen as the most frequent co-morbid among cancer patients followed by Tuberculosis which has been known to contribute to lung cancers [17].

Though the number of smokers encountered in our study was less, a greater number accepted consuming smokeless tobacco. This was an umbrella term summarizing all the basic non-tobacco agents prevalent in our society namely betel nut, areca nuts, *gutka*, *mainpuri*, *bidi*, *naswar* etc. It was not a surprising finding since the worldwide burden imposed by smokeless tobacco is greatest in South Asia with around 100 million consumers in India and Pakistan alone [17]. Multiple roadside cabins providing such hazardous substances at low prices provide easy accessibility and the public has no struggle in purchasing them. This is the reason why we found that after breast cancer, the most commonly reported cancer was head and neck which are strongly linked to consumption of smokeless tobacco. This was also reported previously from registry of province of Punjab, Pakistan [18].

Biomass exposure turned out to be the most ignored and consistent risk factor seen amongst patients. Due to lack of gas and electricity supply in numerous areas of Pakistan and Karachi too, people are compelled to utilize biomass as a fuel to meet day to day needs. Round the globe, this silent killer has been reported to have caused around 4.3 million deaths and has been classified as a probable carcinogen (2A) by the International Agency for Research on Cancer (IARC) because of increased evidence affirming its participation in various cancers [19]. The marked use of biomass as a fuel in today’s modernized era is questionable and places patients at risk of cancer that can be avoided if energy resources are adequately provided in the country.

Our cancer patients shared histories of occupational exposure too. The large number of laborers 78% coincides with our findings of low SES and literacy in the study and supports the fact that majority of workers had an increased contact with hazardous industrial substances. Narrowing down to the garment industry which our subjects were most frequently associated with; dyes, solvents, fiber dust used there have been named to bear carcinogenic potential. Serra et al. report hospitalized bladder cancer patients in Spain to have a history of industrial employment [20] while Elliott et al. found increased mortality due to lung cancers in textile workers of North and South Carolina [21].

Though the impact of family history in a disease like cancer has long been known, an effective method to screen generations and prevent subsequent cases ones hasn’t been devised as yet. 25% of the breast cancer cases are said to occur with positive genetic linkages [22]. A good number of participants reported of having had cancer cases earlier in the family. This number included only those who were aware or could recall. The positive finding was that almost all the relatives received cancer treatments.

Approximately 40,000 females succumb to breast cancer every year in Pakistan and it was the most frequently recorded cancer in our study too. A good number believed that stopping feed early resulted in the disease which coincides with the finding that breastfeeding is thought to decrease the risk for cancer development [23]. Research in 2015 found, hormonal status, nulliparity, late age at first birth, obesity and early menarche as specific risk factors for breast cancer. Patients didn’t demonstrate any knowledge regarding these risk factors. Family history plays a very important role in development of breast cancer and patients with positive family histories should be counseled about regular screening so that future generations can be rescued from developing the disease later on.

Though most of the subjects interviewed named betel nut and gutka as contributors to cancer, this was opposed by the high number of smokeless tobacco consumers included. Moreover, beliefs in mythological causes like black magic and laying the entire cancer burden on fate was popular. It was alarming to document that patients were aware of the cancerous potential of a substance like smokeless tobacco but continued to consume it. This can be seen by the high incidence of head and neck cancers encountered in our study. Therefore, large scale efforts should be made to eliminate availability of such cancer causing substances and cessation program should be established at government level.

The recent advancements have managed to increase the number of survivors. But studies prove there is a significant impairment of life function beyond the scope of any treatment modality to address. The disease not only affects the patient himself but caregivers that are also at the risk of developing various disorders, including psychiatric disorders [24]. Patients in our study reported moderate depression and social problems but the financial burden together with the irritation experienced was maximal.

This study can be used as a baseline for further investigation of common cancers in the country and the risk factors contributing to them. However, our study has few limitations: (1) The study reported data from a single tertiary care center of Karachi, Pakistan. (2) This study was conducted in a government sector hospital with majority patients belonging to a low SES and did not include data from the private sector. (3) Another limitation of the study is unequal representations from each age group minimizing our chances to establish a comparison among various age groups. However, JPMC welcomes patients from various ethnicity and areas of Pakistan. This allowed for diversity among our subjects. Though majority of patients belonged to the underprivileged class, JPMC has services like Cyberknife that allow many private hospitals to refer patients here too; hence, a good class comparison could be done. Further nationwide survey is required on regular basis to establish the cancer statistics in Pakistan. Patient education is also important and importantly tobacco cessation facilities should be established by the government to prevent tobacco related morbidity and mortality.

## Conclusion

Our study highlights the prevalence of various cancers among patients at a single tertiary care center in Karachi, Pakistan. Breast cancer is the most prevalent type of cancer present in our population followed by head and neck cancers. It was also found that the local population in our region is mostly aware of the common risk factors that contribute to cancer but fail to avoid them in their daily lives. On the other hand, there were certain individuals who associate cancers with unrelated causes and myths like pollution, black magic, evil eye that tends to deviate attention from genuine risk factors. The financial and psychological stress coming along with the disease warrants behavioral therapies for such patients along with monetary aid so that survivors can resume their daily life much conveniently. Also, quite a few cases were encountered with positive family histories for cancers. It is essential to practice cancer screening at Government setups like Jinnah so that diseases are detected and treated at a much earlier stage. This study can be the first step towards a large scale study. If future efforts are made to control risk factors leading to cancer, this research can be used as a tool for comparison at a small level to check if success has been achieved or not.

## Additional file

Additional file 1: Study questionnaire. (DOCX 50 kb)

## Abbreviations

HBV: Hepatitis B Virus
HIV: Human immunodeficiency virus
HPV: Human papilloma virus
HTN: Hypertension
IARC: International agency for research on cancer
JPMC: Jinnah postgraduate medical centre
SES: Low socioeconomic status
WHO: World health organization
