# Supplementary material for: Cancer and how the patients see it; prevalence and perception of risk factors: a cross-sectional survey from a tertiary care centre of Karachi, Pakistan
Source: BMC Public Health. 2019 Apr 1;19:360. doi: 10.1186/s12889-019-6667-7 (PMC6444817; doi:10.1186/s12889-019-6667-7)

**CANCER PREVALENCE AT A PUBLIC SECTOR HOSPITAL OF A DEVELOPING COUNTRY**

**Age:** _________

**Sex:** Male _____, Female _____ (if female) Reached menopause: Yes ____ No ____

**Marital status**

Married ____ Single ____ Divorced ____ Separated ____ Widowed ____ Not known ____

**Occupation**: _________________________________

**Nationality:** Pakistan ____ Afghanistan ____ Others ________________________

**Ethnic group:** Sindhi ____ Balochi ____ Pathan ____ Punjabi ____ others _________________

**Socio Economic Status:**

Upper Class: 60,000/- to 2,40,000/ Yes ____ No ____

Middle Class: 20,000/- to 60,000/ Yes ____ No ____

Lower Class: 12,000/- to 20,000/ Yes ____ No ____

Poor Class: 6,000/- to 12,000/ Yes ____ No ____

Extremely poor: Less than 6000/- Yes ____ No ____

Unemployed Yes ____ No ____

**Education level:** Illiterate _____ Primary ____ Secondary _____ Graduate ____ PostGrad ____

**Does the patient know about his/her cancer?** Yes _________ No _________

**Data collected from patient:** Yes _____ No _____

**Data Collected from attended :** Yes _____ No _____( If yes Relation with patient-------------------)

**Co-Morbids:**

DM: Yes ____ No ____ Since _____________________

HTN: Yes ____ No ____ Since _____________________

IHD: Yes ____ No ____ Since _____________________

COPD: Yes ____ No ____ Since _____________________

ILD: Yes ____ No ____ Since _____________________

TB: Active _____ Past ____ Diagnosed when? ____________

CRF: Yes _____ No ____ Since ____________________

HEPATITIS: B _____ C _____ No_____ Diagnosed when? _____________________

HIV STATUS: Positive _____ Negative _____ Since ____________________

Others: ____________________________________________________________

**Assesment of Risk factors:**

1. Smoking:

Have you ever smoked? Yes _____ No _____ Since _______yrs Pack years ___________

Current smoker ______ Ex-smoker _______

If Current Smoker: Daily _____ Weekly _____ Monthly _____ More than monthly ______

1. Smokeless tobacco: Yes _____ No ______

Type of smokeless tobacco _____________ No of yrs/Since ____________

1. Alcohol: Yes ____ No ____

Per day consumption ______________ No of yrs/Since _______________

1. Betal Nut/Sweet Supari: Yes ____ No ____ No of yrs/Since _________

Name of Packet _____________

Any Others: ____________________________________________________________________

1. Biomass Exposure**:**

Have you been exposed to extensive wood/coal burning? Yes ___ No ____

No of exposure years: _____________________________

1. Industrial/Occupational (Chemicals/Metals/Wood) Exposure:

Are you linked to any chemical or metal industry? Yes ___ No ____

Are you linked to any power plant? Yes ____ No ____

Are you linked to any woodwork or wood industry? Yes ____ No ____

What industry/plant? _________________

If more than one: ________________________________________________________

Chemical/ Metal exposed to: ______________________

If more than one: _________________________________________________________

Please specify the way you are linked/ Designation: ______________________________

Exposure per day: ________________ (close contact/mild contact/rarely mostly office work)

Since when have you been linked to this industry? ________________

Any other fellow worker has been diagnosed cancer? Yes ____ No ____

Type of manufacture at company/factory: _________________________________________

1. Pesticide/Herbicide Exposure:

History of working at a farm: Yes ____ No _____

OR Industrial exposure to pesticides/herbicides: Yes ____ No _____

Extent of exposure per day: ______________________

Working here since: ______________________

Any other fellow worker has been diagnosed cancer? Yes ____ No _____

1. Radiation Exposure:

Have you been radiated prior to this cancer? Yes ____ No _____

How many times? ___________ Duration since radiated: ___________________

Were you counseled with respect to the adverse effects of radiation? Yes ____ No _____

1. Sunlight Exposure:

Does your occupation require extensive UV/sunlight exposure? Yes _____ No ______

Extent of exposure: __________________

Working for this extent in sunlight since: ___________________

**Tumor** (Please specify the primary site /organ of origin and exact location)

**Date of Diagnosis/ Diagnosed since (days/mths/wks/yrs)**: _________________________

**Primary Site of tumor**: ______________________________________

**Histopathology**: _______________________________________

**How did you get to know about your cancer?**

1. By yourself: Yes ______ No _______
2. Relative Advise: Yes ______ No _______
3. By GP /Hakeem: Yes ______ No _______
4. By Hospital doctor: Yes ______ No _______
5. Any other: _______________________________________________________

**Extent of Disease (FOR SOLID MALIGNANCIES ONLY):**

Localized: Yes ______ No _______

Local extension only: Yes ______ No _______

Local extension plus regional lymph nodes: Yes ______ No _______

Regional Nodes: Yes ______ No _______

Distant Metastasis: Yes ______ No _______

Not known: Yes ______ No _______

Can’t be assessed: Yes ______ No _______

**Treatment received:** (check one or more as applicable)

Surgery: Yes ______ No _______

Chemotherapy: Yes ______ No ______

Radiation: Yes ______ No ______

Hormones: Yes ______ No ______

Palliative care: Yes ______ No ______

Targeted therapy like EGFR: Yes ______ No ______

Others: _____________________________________________________________

Not known: _____________

**Date of starting treatment**: _____________________________________________

Time interval between diagnosis and start of treatment: ______________________________

How much money have you spent on your treatment so far? __________________________

Does financial limitation stand as a hindrance to further treatment? Yes _____ No _____

**Family History of Cancer:**

1. Has any family member been diagnosed cancer too? Yes _____ No _____

Age when diagnosed: __________, current age: ___________

Cancer of: _____________________

Relation with the member: ______________________

What cause has been linked to his/her cancer? _______________________________

Is that member on cancer treatment? Yes _____ No ______

If more than one: ___________________________________________________________

_________________________________________________________________________

1. Did any family member die to cancer? Yes ____ No ____

Cancer of: _________________________

Age when diagnosed: __________, age at death: ___________

Relation with the member: _________________________

Did that member receive treatment? Yes _____ No _____

What cause was linked to his/her cancer? _____________________________________

If more than one: _____________________________________________________________

___________________________________________________________________________

**Patient Education and Misconceptions Regarding Cancer:**

Name 3 most common risk factors for cancer: 1)________2)__________3)_________

Do you believe the following substances lead to cancer development and risk?

1. Smoking Yes _______ No ________
2. Betel Nut Yes _______ No ________
3. Gutka Yes _______ No ________
4. Alcohol Yes _______ No ________
5. Drug Addictions Yes _______ No ________
6. Family History Yes _______ No ________
7. Others---------------------

Do you believe that the following can be linked to cancer development and risk?

1. Fever Yes _______ No ________
2. Injuries Yes _______ No ________
3. Unhealthy diet Yes _______ No ________
4. Tooth extraction Yes _______ No ________
5. Stress Yes _______ No ________
6. Infection Yes _______ No ________
7. Hot Climate Yes _______ No ________
8. Pollution Yes _______ No ________
9. Black Magic Yes _______ No ________
10. Evil Eye Yes _______ No ________
11. Fate Yes _______ No ________
12. Others---------------------

For Female patients:

1. Menstrual Problems Yes _______ No _______
2. Domestic Violence/Beating Yes _______ No _______
3. Stopping Breast Feeding Yes _______ No _______
4. Others-------------------

Do you think it is possible to cure cancer? Yes _____ No _____

Do you think it is possible to cure cancer in a public sector hospital? Yes _____ No ______

**During the past 2 weeks:**

I have felt anxious or worried about cancer and the treatment I am receiving.


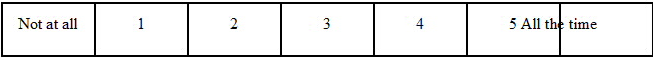


I have felt depressed or discouraged.


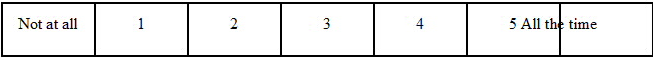


I have been irritable or unusually angry, and I have not controlled it well.


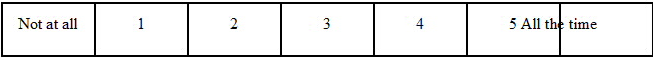


I have noticed a change in my appetite.


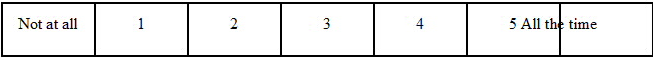


I have had trouble focusing at work or at home, or on routine things such as reading the newspaper or watching television.


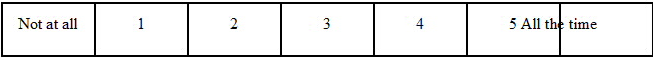


Cancer and its treatment have interfered with my daily activities.


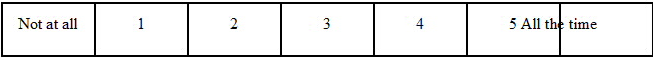


Cancer and its treatment have interfered with my family or social life.
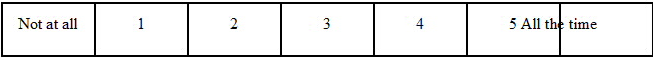


Cancer has caused physical, emotional, or financial hardship for me.


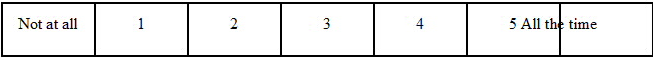


Cancer and its treatment have caused changes in how I look, and this concerns me.


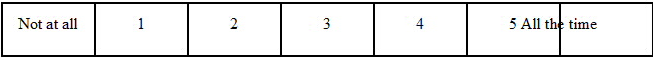


I have had trouble coping with the stress I have been having.


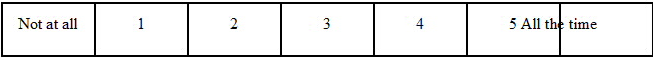


My quality of life during the past 2 weeks has been:


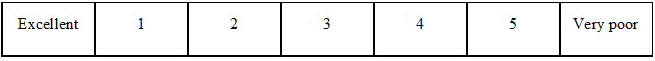

Supplement: Supplementary file 1 — Study questionnaire. (DOCX 50 kb) [file 12889_2019_6667_MOESM1_ESM.docx]
